# Central regulation of breast cancer growth and metastasis

**DOI:** 10.20517/2394-4722.2018.107

**Published:** 2019-03-28

**Authors:** Jeremy C. Borniger

**Affiliations:** Department of Psychiatry & Behavioral Sciences, Stanford University School of Medicine, P154 MSLS Building, 1201 Welch Rd., Stanford, CA 94305, USA.

**Keywords:** Breast cancer, hypothalamus, immunometabolism, sympathetic nervous system, neuromodulators

## Abstract

Cancer is a systemic disease. In order to fully understand it, we must take a holistic view on how cancer interacts with its host. The brain monitors and responds to natural and aberrant signals arriving from the periphery, particularly those of metabolic or immune origin. As has been well described, a hallmark of cancer is marked disruption of metabolic and inflammatory processes. Depending on the salience and timing of these inputs, the brain responds via neural and humoral routes to alter whole-body physiology. These responses have consequences for tumor growth and metastasis, directly influencing patient quality of life and subsequent mortality. Additionally, environmental inputs such as light, diet, and stress, can promote inappropriate neural activity that benefits cancer. Here, I discuss evidence for brain-tumor interactions, with special emphasis on subcortical neuromodulator neural populations, and potential ways of harnessing this cross-talk as a novel approach for cancer treatment.

## INTRODUCTION

Uncovering the relationships among cancer and the physiology of its host has cemented the notion that cancer is a systemic disease. Cancer patients frequently experience systemic symptoms like depression, sleep disruption, cognitive impairment, appetite and metabolic dysfunction, and weight loss. These phenomena span different cancer types and occur independently from treatment regimens. Clinical studies consistently report that such symptoms (such as weight loss, sleep disruption, and circadian misalignment) are predictors of poor prognoses and reduced quality of life^[1-5]^. Tumors are capable of altering local macronutrient contents that modulate infiltrating immune cell function resulting in aberrant inflammation. Additionally, they secrete metabolic “waste”, which can promote inflammation and alter the function of distal organs and tissues such as the liver and brain^[6-10]^. As evidence accumulates, we are learning that many of these cancer-associated co-morbidities are (at least in part) due to deregulation of normal brain function by the cancer itself, cancer treatment(s), or other factors.

**Figure 1.**
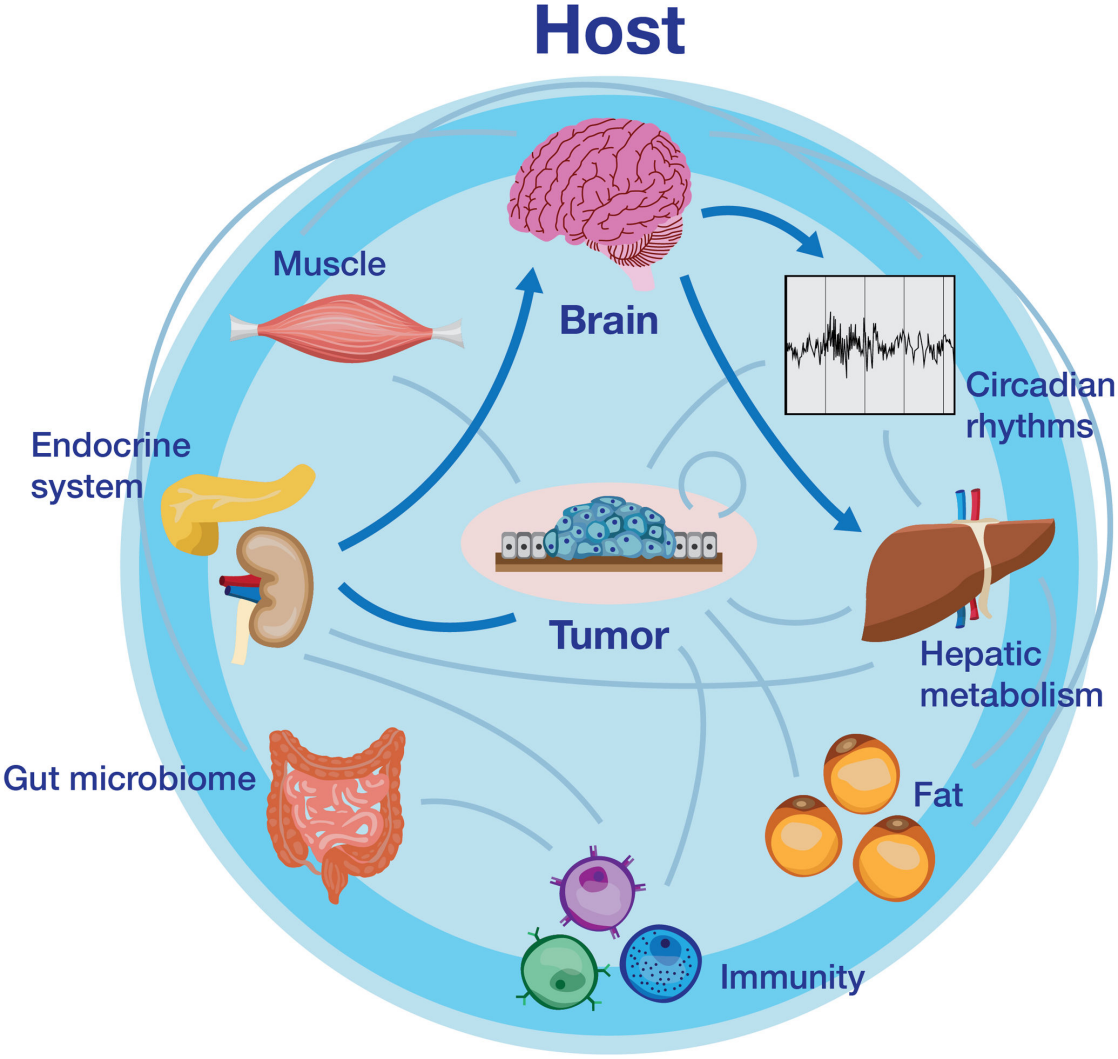
A simplified schematic of reciprocal tumor-host interactions. Tumors promote aberrant physiology via alterations to the immune system and secretion of metabolic “waste” which contributes to further inflammation and altered function of distal organs, including the brain. Feedback from the brain (neural or humoral) can subsequently exacerbate tumor-associated immune and metabolic changes, ultimately facilitating tumor growth, angiogenesis, metastasis, or cancer-associated co-morbidities

Reciprocally, the host system can influence tumor growth and metastasis via immune, endocrine, and neural pathways. For example, chronic stress, which results in dysregulation of glucocorticoid and adrenergic signaling, exacerbates tumor growth and angiogenesis^[11,12]^. Additionally, chronic sleep fragmentation, resulting in top-down impairments to the immune system, further promotes tumor growth^[13]^. The objective of this review is to provide an up-to-date overview of cancer as a systemic disease from a basic science perspective [Figure 1]. Special focus will be given to subcortical neural populations that are sensitive to signals arriving from peripheral tissues and the environment, as well as those that send long-range projections to modulate immune or metabolic function, ultimately facilitating cancer growth and/or metastasis. Through understanding these brain-tumor interactions, potential undescribed drug or lifestyle targets will be uncovered. Additionally, these studies would open up space for existing therapies to be repurposed for effective cancer treatment (as is the case with the anti-obesity drug Metformin^[14,15]^.

## NEURAL CIRCUITRY DEREGULATED IN CANCER

### Sleep disruption

Disruption of sleep and/or circadian rhythms in physiology and behavior are frequently observed in cancer patients. Indeed, 35%-80% of cancer patients report poor sleep quality^[16,17]^, as compared to 29%-32% of the general population^[18]^ [Table 1]. These problems may stem from the cancer itself, the stress or stigma surrounding a cancer diagnosis, different treatment regimens (e.g., chemotherapy, immunotherapy and/or radiotherapy), or additional lifestyle factors^[19]^. These problems are prevalent across a variety of cancer types, with lung and breast cancer patients making up the majority of the population experiencing these symptoms^[2,20-22]^. A “chicken-or-the-egg” phenomenon has emerged: poor sleep associates with elevated cancer incidence and progression, and cancer and/or cancer treatments further promote sleep disturbance^[2,3,6,23]^. Due to the heterogeneity among cancer types, patient populations, treatment regimens, and lifestyle factors, it has been challenging to pin down cause and effect. This lack of knowledge prevents targeted therapies from being developed and impairs quality of life and lifespan in cancer survivors. For example, sleep disruption is associated with increased mortality in breast cancer independent of other factors like estrogen receptor status, depression, anxiety, and socioeconomic status^[3]^.

The hypothalamus is a critical structure for maintaining homeostasis^[24,25]^. Although beyond the scope of this review, a brief discussion of its relevant circuitry is warranted to put the rest of our discussion in context. Its functions include the regulation of sleep-wake cycles, circadian rhythms, body temperature, feeding/metabolism, the stress response, and reproduction, among others. Many of these are linked to either the promotion of cancer development or it’s progression (as I discuss in subsequent sections). The lateral hypothalamus (LH) is a highly heterogeneous structure that serves a primary role in arousal, metabolism, and motivated behavior^[24]^. A neural population that has been intensely studied in this area are those that express the excitatory neuromodulators hypocretin-1 and hypocretin-2 (aka orexin-A and -B; HO)^[26,27]^. Discovered by two groups at essentially the same time^[28,29]^, these neurons are critical for maintaining wakefulness, as their destruction results in the sleep disorder narcolepsy^[30-33]^.

HO neurons project throughout the brain to participate in functions ranging from arousal and motivation, to anxiety and reproductive behavior^[27]^. Importantly, they also send long range projections that modulate sympathetic outflow from the brain^[34]^. Indeed, disinhibition of HO neurons in the LH can directly influence hepatic gluconeogenesis, promoting *de novo* glucose production upon stimulation^[35]^. Reciprocally, HO neurons are sensitive to metabolic signals arriving from the periphery. These include hormones and other messages important in cancer regulation, including leptin, ghrelin, glucose, dietary amino acids, and changes in extracellular pH and CO_2_ concentrations^[27]^. Stimulation of HO neurons further activates the hypothalamic-pituitary-adrenal (HPA) axis, resulting in rapid increases in circulating glucocorticoid concentrations^[36]^. Aberrant glucocorticoid rhythms are highly prevalent in breast cancer patients^[4]^, and their actions on the immune system may influence patient prognosis (discussed below).

Leptin, an adipokine hormone that correlates with satiety and body fat accumulation, generally inhibits HO neurons through direct and indirect pathways^[36-38]^. Specifically, intermingled neurons expressing the long-form leptin receptor (LepRb) provide direct inhibitory input to HO neurons. Overexpression of leptin or it’s cognate receptor (Ob-R) in mammary tumors and nearby normal epithelial cells is associated with progressive and metastatic breast cancer^[39,40]^. In this way, leptin overexpression may be relevant to fatigue and sleep disruption in cancer patients, through its inhibitory actions on HO neurons. Ghrelin, an orexigenic hormone produced primarily in the stomach^[41-43]^ has an excitatory effect on HO neurons, and inhibition of HO neural activity can prevent ghrelin-induced feeding behavior^[44,45]^. Ghrelin or the activity of its catalytic enzyme ghrelin-O-acyl-transferase is frequently deregulated in cancer^[46-48]^, where it associates with cancer-induced cachexia. The role these and other metabolic factors play in cancer and cancer-related co-morbidities is coming into focus as the research community begins to examine them in addition to long-standing candidates from the immune system such as cytokines [e.g., interleukin (IL)-1β, IL-6, TNF-α] and chemokines (e.g., CCL2, CXCL12).

**Figure 2.**
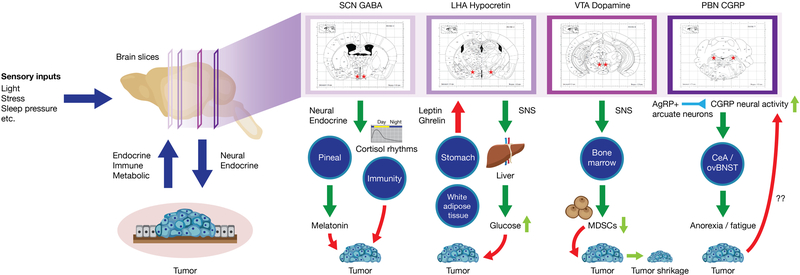
Highlighted pathways linking the brain and periphery in the context of cancer. Environmental (e.g., light, stress) or endogenous signals reach the brain to alter the activity of neurons involved in sleep (LHA hypocretin/orexin), circadian rhythms (SCN-GABA), reward (VTA-Dopamine), metabolism, and energy balance (Parabrachial CGRP). Aberrant activity of these cells promotes signaling in the periphery that ultimately facilitates tumor growth, angiogenesis, and invasiveness. Systems highlighted are bolded in Table 2. LHA: lateral hypothalamic area; SCN: suprachiasmatic nucleus; GABA: gamma amino butyric acid; VTA: ventral tegmental area; PBN: parabrachial nucleus; CGRP: calcitonin gene related peptide; MDSCs: myeloid derived suppressor cells; SNS: sympathetic nervous system; AgRP: agouti related peptide

Indeed, brain-tumor-metabolic interactions were recently tested in a mouse model of non-metastatic breast cancer^[6]^. Borniger, Walker and colleagues examined sleep and whole-body metabolic changes during the course of tumor progression. They observed marked peripheral inflammation driven by the cytokine IL-6. This was associated with a shift towards hepatic gluconeogenesis over glycolysis in tandem with disrupted sleep [Figure 2]. Additionally, tumor-bearing mice had reduced circulating leptin concentrations and were hypersensitive to the orexigenic hormone ghrelin. As HO neurons are sensitive to these peripheral metabolic signals, and they are powerful regulators of wakefulness, the authors examined whether their activity was modulated by tumor growth. They noted that tumors promoted aberrant activity within HO neurons, and inhibition of their signaling (via administration of a dual HO receptor antagonist) attenuated both metabolic and sleep problems. The authors reasoned that in order for HO neurons to influence peripheral glucose metabolism, a signal must reach the liver from the brain. A potential pathway through which this could occur is the sympathetic nervous system (SNS)^[35]^. Ablating the SNS with administration of 6-hydroxydopamine (6-OHDA) rescued tumor-induced metabolic deficits, supporting the idea that HO neurons modulate peripheral glucose concentrations via downstream SNS activation^[6]^. Significantly more research is required to unravel the complex signaling network linking tumors in the periphery to changes in the activity of this critical neural population. However, these findings suggest that repurposing drugs targeting this system [e.g., Suvorexant (Belsomra^®^)] may be a novel strategy for improving sleep and metabolic health in patients with cancer.

In two mouse models of lung cancer (LLC and TC1), Hakim *et al*.^[13]^ demonstrated that chronic sleep fragmentation promoted tumor growth, a phenotype that was abolished in mice lacking the endotoxin receptor TLR4. TLR4 is part of a family of pattern recognition receptors that powerfully engage the innate immune system upon ligand binding. Surprisingly, the effect of sleep fragmentation on tumor progression was maintained in mice lacking TLR4 effector molecules MyD88 or TRIF, although the effect was reduced. This was the first study to causally link disrupted sleep, tumor progression, and immune deregulation. Although this approach lacks cell-type specific investigations into neural populations influenced by the sleep fragmentation protocol, it suggests one or more neural populations sensitive to this manipulation may be responsible for top-down changes to the immune system that biases the host environment to one that favors tumor growth. Recently, McAlpine *et al*.^[49]^ demonstrated one such pathway, where sleep disruption decreased the number of HO-expressing neurons. This led to aberrant regulation of pre-neutrophils in the bone marrow, which were found to express HO receptor 1. This change in activity promoted egress of myeloid lineage cells, which then contributed to the development of atherosclerosis. I speculate that a similar phenomenon occurs in the context of cancer^[49]^. The studies discussed in this section highlight the bidirectional pathway between sleep and cancer, where disrupted arousal influences cancer growth and aberrant neoplasia reciprocally promotes changes in sleep.

### Circadian deregulation

The paired suprachiasmatic nuclei (SCN) are the primary structures responsible for setting circadian rhythms in physiology and behavior that we observe across most of the phylogenetic tree^[50-53]^. The SCN receive photic input from specialized retinal ganglion cells that serve a minimal role in vision. These cells express a photosensitive protein, melanopsin, allowing them to directly sense light, and are therefore named “intrinsically photosensitive retinal ganglion cells” (ipRGCs)^[54,55]^. ipRGCs transduce photic input into a neurochemical one, with axons traversing the retino-hypothalamic tract and terminating in the suprachiasmatic nucleus. Here, glutamate-mediated synaptic transmission results in downstream cyclic adenosine monophosphate (cAMP) accumulation and cAMP response element binding (CREB) phosphorylation. Phosphorylation of CREB results in it binding the promoters of the core clock genes *per* and *cry*. In a transcription-translation loop, the protein products homo or heterodimerize (e.g., PER::CRY dimers), enter the nucleus, and suppress the transcription of the positive arms of the circadian clock, the genes *arntl1 (bmal1)* and *clock*.

This process takes approximately 24 h to complete, where light-induced gene transcription has a phase-modulatory effect on the clock. This feedback loop operates in a cell-autonomous manner throughout the body, with peripheral clocks “set” via neural and humoral routes originating from the SCN^[56,57]^. Behavioral and physiological outputs controlled by the clock include sleep-wake cycles, appetite and food intake, mating and reproductive behavior, rhythms in immune function and glucocorticoid secretion, and stress responses, among others.

Chronic circadian disruption (e.g., via aberrant light exposure, genetic manipulations, or phase shifting) is repeatedly associated with spontaneous cancer occurrence in humans and multiple rodent models spanning a variety of cancer types^[2,4,21,58,59]^. For example, chronic circadian disruption via repeated inversions of the light-dark cycle promotes spontaneous tumor development in a mouse model of breast cancer mimicking Li-Fraumeni syndrome^[60]^. This paradigm is known to cause significant disruption of the circadian clock as well as the sleep-wake cycle. Using a transgenic approach to specifically knockdown the tumor-suppressor p53 in mammary epithelial cells (*WAP-Cre::p53^fl/fl^*), van Dycke and colleagues demonstrated that mice undergoing the inversion protocol developed mammary tumors ~8 weeks sooner (median; 17% sooner) than their control counterparts. This was accompanied by increased body mass gain in mice experiencing circadian disruption, as well as gross increases in sleep throughout the experiment. This was the first study to demonstrate a causal role for light-induced circadian disruption in the acceleration of spontaneous breast cancer development.

In a similar study, Papagiannakopoulos and colleagues investigated the effects of environmental and genetic circadian disruption on lung tumorigenesis^[61]^. Using a cre-inducible model of lung cancer [*K-ras^LSL-G12D/+^;*p53*^flox/flox^* *(KP) mice*], the authors subjected the mice to a jet-lag circadian disruption schedule and examined tumor growth, metabolism, and proliferative capacity. Chronic jet-lag accelerated tumor growth, severity, and mortality upon cre-mediated recombination. A similar phenotype was uncovered when manipulations consisted of knocking out core clock genes (*Per2* or *Bmal1 (Arntl1)*) in animals that develop spontaneous cancer (*Kras*^*LA2*/+^ mice). Tumor cells deficient in *per2* were also more proliferative in culture, and mouse embryonic fibroblasts lacking *kras* and *per2* were more sensitive to cellular transformation than their Per2-intact counterparts. As energy balance is powerfully regulated by circadian rhythms, they investigated cellular metabolic pathways in Per2 deficient cells. Indeed, cells lacking this core clock gene showed a marked increase in the excretion of core energy substrates lactate, glucose, and glutamine, indicating a systemic effect of circadian disruption. Using isotope labeled glucose (U-13Cglucose) and carbon 4 (M4) labeling they demonstrated that cells with disrupted circadian clocks increased the amount of glucose loaded into the tricarboxylic acid cycle, a finding that agreed with prior reports^[62]^. Finally, they investigated whether clock gene abnormalities were found in primary patient tumors and noted that all genes (except for *clock*) were down-regulated in lung cancer samples.

In a reciprocal set of experiments to those discussed above, Masri & colleagues investigated how tumors themselves disrupt host circadian rhythms, independent of the outside environment^[10]^. In a mouse model of lung adenocarcinoma, they demonstrated that tumors dysregulated the circadian expression of genes controlling immunity and metabolism in a distal organ, the liver, without affecting core components of the circadian clock. This was subsequently confirmed in an additional model of non-metastatic breast cancer, as discussed above^[6]^. These changes were hypothesized to be due (in part) to tumor-induced IL-6 signaling interfering with insulin-dependent glucose uptake via a SOCS3-regulated mechanism. Experiments like those discussed above highlight the bidirectional cross-talk among the circadian system (ultimately controlled by the brain), tumors, and the host. These findings suggest that novel approaches for cancer treatment lie in the normalization of circadian rhythms via light, nutrition, or clock phase or amplitude-modulating compounds. Indeed, a flavonoid found in citrus peel, nobiletin, is a powerful clock-enhancing molecule^[63]^ that shows promise in the treatment of a variety of cancers^[64-66]^.

### Melatonin

Melatonin is an indoleamine hormone produced and secreted into circulation primarily by the pineal gland in mammals, where it acts as an endogenous signal of darkness^[56,67-70]^. Through a poly-synaptic pathway, the suprachiasmatic nuclei control melatonin production and secretion, rendering the concentrations of this hormone sensitive to environmental light input^[71]^. Because light activates the SCN to cause downstream inhibition of the pineal gland, darkness induced disinhibition permits melatonin secretion only during the night.

Melatonin is a pleiotropic immunomodulatory molecule. Broadly, melatonin is immune-enhancing, acting as a mild anti-inflammatory agent, buffering the immune system against glucocorticoids and reactive oxidative and nitrosative stress^[72-74]^. Shift work and transmeridian travel, two behaviors that strongly alter melatonin rhythms, are associated with cancer incidence. In 2007, the International Agency for Research on Cancer classified shift work with circadian disruption or chronodisruption as a probable human carcinogen^[75]^. Artificial light at night (e.g., street and house lights), which inhibits pineal melatonin, is associated with increased breast cancer prevalence^[22,76,77]^, although the findings are not universally consistent^[78]^. The mechanisms behind these trends are becoming clearer thanks to basic research.

In a clever experimental design, Blask & colleagues investigated the role of melatonin on human breast cancer xenograft tumor progression in nude rats^[79]^. Blood samples were collected from healthy female volunteers during the day, night, or after 90 min exposure to bright white light at night (to putatively knockdown circulating melatonin concentrations). Melatonin deficient- (daytime or light at night collected) or sufficient blood were then perfused into the tumor xenografts. Tumors perfused with daytime or light at night-exposed blood samples showed high proliferative activity and linoleic acid uptake/metabolism, while those perfused with melatonin-rich nocturnal blood had markedly reduced proliferative activity. Additionally, exposing tumor-bearing rats to increasing intensities of artificial light dose-dependently accelerated tumor growth in tandem with knockdown of circulating melatonin. These results were the first to suggest that light at night exerts its pro-tumorigenic effects via its actions on circulating melatonin concentrations^[79]^. Since the publication of this study, melatonin has been intensely investigated as an anticancer molecule, particularly in the context of breast cancer^[80,81]^. Potential mechanisms for its actions have been uncovered, including antiestrogen, angiogenic, and oxidant pathways^[82]^. As an ancient and pleiotropic hormone, melatonin is not the “cleanest” anti-cancer molecule, given its distributed effects on many tissues throughout the body. However, understanding the mechanisms by which it exerts its anti-cancer effects will likely lead to novel and targeted treatments^[83]^. Additionally, due to its low toxicity and high tolerability, it may be useful as a powerful and inexpensive adjunct therapy.

### Midbrain reward system

The midbrain ventral tegmental area (VTA) and neighboring substantia nigra are the primary source of all dopamine (DA) within the brain. Known for its important role in reward and motivational processing (i.e., calculating reward-prediction errors), the VTA has recently become a target for modulating cancer. Elevated concentrations of dopamine are associated with blunted tumor growth, reduced angiogenesis, and lower metastatic capacity of cancer in rats^[84]^. In general, dopamine seems to inhibit cancer growth, while serotonin facilitates it^[85]^. The mechanisms underlying this phenomenon are unclear, although research has started to make headway in this area. In recent years, the VTA has been linked to the modulation of both innate and adaptive immunity^[86]^. Using designer receptors exclusively activated by designer drugs (DREADDs), Rolls and colleagues demonstrated that activation of VTA-DA neurons promotes monocyte/macrophage expansion and innate immune responses to *E. coli* infection. Activation of these neurons further increased the number of circulating B-cells, subsequent IgM and IgG titers in response to *E. coli*, and interferon-g production by T-cells, suggesting enhanced adaptive immunity.

After these initial studies, they applied their findings to a mouse model of lung cancer^[87]^. After injecting viruses encoding Gq-coupled DREADDs into the VTA (as previously), mice were injected with subcutaneous tumor cells (LLC or B16 cancer cells), and then given daily injections of the DREADD ligand CNO, chronically activating the VTA. Mice that were “VTA-activated” developed smaller tumors than control mice that did not express the DREADD in the VTA [Figure 2]. To examine how this signal from the brain might reach the tumor, the authors ablated the sympathetic nervous system using the neurotoxin 6-hydroxydopamine (6-OHDA; as discussed earlier). Mice that were SNS-ablated (or received a beta-adrenergic receptor antagonist) failed to reduce their tumor burden upon VTA-DA activation. They further showed that VTA activation altered norepinephrine concentrations specifically in the bone marrow, a vital immune compartment. This strongly supports the hypothesis that VTA-DA neurons alter tumor growth via SNS innervation of the bone marrow. As myeloid derived suppressor cells (MDSCs) express beta-2 adrenergic receptors and regulate tumor growth via inhibition of anti-tumor immunity, the authors examined their phenotype in response to VTA activation. DREADD-induced VTA activation reduced the number of MDSCs, suggesting that the actions of central VTA stimulation on tumor growth may be through sympathetic suppression of MDSCs. To test the role these cells played in their model, they adoptively transferred MDSCs from VTA-activated mice to control mice not expressing the Gq-coupled DREADD in the VTA. This recapitulated the anti-tumor effect of VTA-activation. This suggests that modulation of the immune system via a discrete population of neurons within the brain acts (at least in part) to suppress tumor growth via the sympathetic nervous system.

### Stress - glucocorticoids and catecholamines

Glucocorticoids (primarily cortisol in humans and corticosterone in mice) are powerfully regulated by circadian rhythms, stress, metabolic state, and immune status^[88]^. Their production and regulation along the HPA-axis has been known for several decades. Their role in linking psychological stress to cancer, however, has only become a subject of intense research within the 21st century^[11]^. First hinted at in the 70’s and 80’s, psychological stress has been suspected to influence tumor growth for several decades^[89,90]^. Their role in cancer associated metabolic stress is more well defined. For example, upon metabolic stress induced by cancer-related inflammation (impairments in ketogenesis), glucocorticoids can act to suppress anti-cancer immunity^[91]^. This is associated with disrupted rhythms in glucocorticoid secretion, a component controlled ultimately by a crosstalk between central clocks in the SCN and ancillary oscillators in the adrenal glands^[92]^. Adrenergic signaling, largely driven by activation of the SNS in the context of stress, also has immunomodulatory properties (as discussed above).

Thaker, Sood & colleagues provided empirical evidence that psychological stress can facilitate tumor growth in multiple animal models via its promotion of glucocorticoid and adrenergic signaling^[12,93]^. These studies demonstrated that multiple ovarian cancer tumor cell lines (e.g., EG, SKOV3, 222, HeyAs…) enhance invasiveness when exposed to norepinephrine and/or glucocorticoids (in part) via the upregulation of matrix metalloproteinases (MMPs), critical regulators of angiogenesis and tissue remodeling. Blockade of adrenergic signaling or inhibition of MMPs prevented elevations in cell invasiveness. *In vivo* experiments demonstrated that chronic behavioral stress (restraint) increased tissue catecholamines, tumor growth, vascularization, and invasiveness in an orthotopic mouse model of ovarian cancer. These effects were driven by adrenergic signaling (through the b2-adrenoceptor), resulting in downstream cAMP-protein kinase A (PKA) pathway activation. This subsequently promoted the transcription of vascular endothelial growth factor and the MMPs (−2 and −9). These findings highlight adrenergic-receptor signaling as a potential target for reducing tumor angiogenesis and growth. Indeed, perioperative cyclo-oxygenase 2 and beta-adrenergic blockade was shown to improve measures of metastasis in breast cancer patients, offering a safe and effective adjuvant treatment strategy^[94]^.

### Energy balance and feeding

Disrupted energy balance resulting in enhanced capacity to sustain proliferative growth is a hallmark of cancer^[9]^. Indeed, one of the first major breakthroughs in cancer research was the discovery that tumor cells are biased towards aerobic glycolysis rather than oxidative phosphorylation to produce energy (i.e., the Warburg effect^[95-97]^). Therefore, a common finding in malignant cancers is a strong upregulation of lactate and catalytic enzymes required for lactate production from pyruvate (e.g., lactate dehydrogenase/^[98-101]^. Lactate normally acts to aide in glucose sensing and food intake, where it is transported into the brain via monocarboxylate transporters present on endothelial cells lining the blood-brain barrier^[102]^. After entering the brain, lactate is able to interact with neurons that normally promote food intake, such as those that produce agouti-related peptide (AgRP) within the arcuate nucleus. Lactate’s mechanism of action on orexigenic cells is via its effects on the adenosine monophosphate kinase/methylmalonyl CoA signaling pathway within the hypothalamus^[103]^. Lactate alone, however, does not seem to be responsible for cancer-associated anorexia (discussed below)^[104]^.

Anorexia is a common phenomenon in cancer patients with weight loss, and even when patients attempt to eat enough to compensate, they frequently cannot maintain a healthy weight. Although significant evidence suggests that inflammatory signaling secondary to tumor growth or cancer-treatment associates with anorexia, a specific neural population and mechanism governing this common problem is lacking^[99]^. An attractive candidate neural population that may underlie these traits (in part) is the calcitonin-gene-related-peptide (CGRP) expressing population of cells in the parabrachial nucleus (PBN_CGRP_). These cells powerfully suppress appetite and promote the termination of feeding behavior^[105,106]^. CGRP neurons are activated by upstream circuits that respond to cancer-associated signals, and are inhibited by those that promote feeding, including hypothalamic AgRP/neuropeptide Y neurons^[107]^. These neurons are also sensitive to peripheral noxious and painful stimuli, which are other aspects of cancer progression^[108]^.

In a mouse model of Lewis lung carcinoma, Schwartz and colleagues investigated how peripheral tumors modulate CGRP neural activity and their role in cancer-associated anorexia/cachexia^[109]^. CGRP neurons were strongly activated in tumor-bearing mice compared with controls, a phenotype typically found after ingestion of a large meal. This suggests that tumors activate cells normally responsible for meal termination and cessation of feeding behavior. Using a cre-dependent tetanus toxin transgene, they demonstrated that inactivation of these cells prevented cancer-associated anorexia/cachexia. Additionally, this manipulation normalized the activity of neurons in circuits downstream from the PBN, namely the central amygdala (CeA) and oval subnucleus of the bed nucleus of the stria terminalis (ovBNST), which may play additional roles in cancer-associated behavioral phenotypes. To control the activity of PBN_CGRP_ neurons with better temporal precision, they used Gi-coupled DREADDs to transiently inhibit these neurons in anorexic/cachexic mice. This manipulation was able to recapitulate the effects seen with their previous approach using tetanus toxin.

Another research area that is rapidly growing in scope is that of brain-gut and gut-cancer interactions. Changes in systemic microbial diversity can influence brain function, alter immune phenotypes, and dictate subsequent cancer development or a tumor’s response to immunotherapy^[110,111]^. In a proof-of-principle experiment, Lakritz *et al*^[112]^ demonstrated that *Helicobacter hepaticus*, a pathogenic gut microbe, promotes distal breast tumorigenesis in a neutrophil-dependent manner. Cancer-prone female mice (FVB-Tg(C3-1-TAg)cJeg/JegJ) were infected with *H. hepaticus* (via gastric gavage) at 3 months of age, and then assessed for subsequent mammary tumorigenesis. Mice infected with these bacteria developed significantly more tumors than their counterpart controls that were not infected. Additionally, mammary intraepithelial neoplasias were associated with strong neutrophil invasion (myeloperoxidase staining). Chronic depletion of neutrophils (via anti-Ly6-G antibodies) prevented *H. hepaticus*-induced cancer development. These data suggest that host-microbe interactions may drive cancer in distal tissues through an immune-mediated mechanism.

## CONCLUSIONS AND IMPLICATIONS

Together, the studies discussed above aim to provide an understanding of the types of inputs the brain receives, the signals it propagates, and the effects of these messages on tumor growth and metastasis. Reciprocally, tumor-induced changes in physiology are relayed to the brain via endocrine, immune, or neural signals that ultimately change the activity of discrete neural populations important for maintaining homeostasis. Resolving the “conflict of interest” between cancer and the brain will undoubtedly lead to improvements in patient quality of life and unlock a novel means for cancer treatment. A summary of these findings from basic science are presented in Table 2.

In this vein, treatments targeting the circadian system (i.e., chronotherapy) have gained significant traction in recent years^[113,114]^. These approaches leverage natural circadian rhythms in metabolism and detoxification systems to schedule chemotherapy or radiotherapy to coincide with times of peak effectiveness with the lowest potential for side-effects. Animal models have further demonstrated that this approach can effectively limit hepatic toxicity and the inflammatory response to chemotherapeutics^[115,116]^. Artificially boosting circadian rhythms (e.g., with nobiletin) adds an additional prospective anti-cancer strategy^[64]^.

Alternatively, targeted stimulation of specific brain areas deregulated in cancer may help overcome resistance to more traditional treatment strategies. As discussed above, stimulation of the dopaminergic ventral tegmental area promotes tumor suppression via the sympathetic nervous system^[87]^. If findings such as these translate to humans, deep brain stimulation protocols could be adapted for adjuvant cancer treatment. For example, deep brain stimulation of the subthalamic nuclei for Parkinson’s disease promotes sympathetic activation in a safe and reversible manner^[117,118]^, a procedure that could be repurposed in the context of advanced cancer. Alternatively, biobehavioral therapies can be designed to promote positive thinking and rewarding experiences (to activate the dopaminergic system) to aide in cancer suppression. Indeed, mindfulness meditation has been demonstrated to improve mood, reduce stress, and attenuate inflammation in patients with breast cancer^[119]^.

As cancer drastically alters energy balance, influencing the activity of specific brain nuclei regulating metabolism and food intake (e.g., hypocretin, AgRP, POMC, CGRP neurons) represents a strategy to not only improve quality of life, but limit energy availability to the cancer. Indeed, inhibition of aberrant hypocretin/orexin signaling promotes sleep and attenuates tumor-induced metabolic abnormalities in a mouse model of breast cancer^[6]^. Repurposing drugs that modify food intake and energy balance (e.g., metformin) further provides additional avenues for adjuvant cancer therapy. However, significant more research is needed to understand both (1) how the brain influences cancer-associated immune populations and (2) how the tumor communicates with the brain to deregulate homeostasis and health. Only then can we begin to manipulate this cross-talk to facilitate cancer elimination.

## Figures and Tables

**Table 1.** Non-exhaustive list of clinical observations of systemic co-morbidities potentially influencing brain function (sleep disturbance, circadian rhythm disruption, cognitive impairment, metabolic abnormalities, microbial dysbiosis, and systemic inflammation) in patients with cancer

Systemic problem	Patient population	Methods	Primary observation	Ref.
Sleep disturbance	823 patients with cancer receiving chemotherapy	Post-hoc analysis of data from a large randomized clinical trial; Hamilton Depression Inventory used to assess sleep disturbance	36.6% (*n* = 301) of the patients with cancer reported insomnia symptoms, and 43% (*n* = 362) met the diagnostic criteria for insomnia syndrome; breast cancer had the highest number of overall insomnia complaints	[120]
	85 women with Stages I-IIIA breast cancer	actigraphy for 72 consecutive hours and filled out questionnaires (PSQI, MFSI-SF, FOSQ, FACT-B, and CES-D) on sleep, fatigue, depression, and functional outcome	women slept for ~6 h a night and napped > 1 h during the day. Sleep was disturbed and fatigue levels were high; phase-delayed circadian rhythms	[121]
	97 women with advanced breast cancer (age = 54.6 ± 9.8 years)	72 h actigraphy; sleep efficiency was determined as the ratio of total sleep time to total sleep time plus wake after sleep onset	Sleep efficiency predicted reduction in overall mortality [hazard ratio (HR), 0.96; 95% confidence interval (CI), 0.94-0.98; *P* < 0.001] at median 6 y follow-up. Remained significant (HR, 0.94; 95%CI, 0.91-0.97; *P* < 0.001) after adjusting for age, estrogen receptor status, cancer treatment, metastatic spread, cortisol levels, and depression	[3]
	40 patients (50 years, SD = 11; 53% White, 28% Asian, 19% Other) with primary breast cancer (18% Stage I, 50% Stage II, 33% Stage III) undergoing chemotherapy	Neurocognitive battery of tests including PSQI, ISI, BFI, CAD, COWAT, HVLT; actigraphy for 7 consecutive days to track arousal/sleep	Better circadian function was associated with less sleep disruption (PSQI, *r* = −0.44, *P* = 0.005) and less insomnia (ISI, *r* = −0.42, *P* = 0.008). Both subjective sleep alteration and circadian disruption were associated with levels of fatigue (BFI, all *P*-values < 0.05) and sleep disruption measures were strongly associated with depression and anxiety (ISI: *r* = 0.51, *P* = 0.001; PSQI: *r* = 0.43, *P* = 0.005)	[122]
Circadian rhythm disruption	389 Caucasian cases and 432 Caucasian controls	Investigated the association between an exonic length variation in a circadian gene, Period3 (Per3), and breast cancer risk using blood samples collected from a recently completed breast cancer case-control study in Connecticut	*Per3* genotype (heterozygous + homozygous 5-repeat alleles) was associated with an increased risk of breast cancer among premenopausal women (odds ratio, 1.7; 95%CI, 1.0-3.0)	[123]
	57 presurgical breast cancer patients	Daily self-reports of cancer-specific distress and avoidant coping as well as actigraphic and salivary cortisol data	Distress and avoidant coping were related to rest/activity rhythm disruption (daytime sedentariness, inconsistent rhythms). Patients with disrupted rest/activity cycles had flattened diurnal cortisol rhythms	[124]
	104 patients with metastatic breast cancer	Salivary cortisol levels assessed at study entry at 800, 1200, 1700, and 2100 hours on each of 3 consecutive days; NK cells measured using flow cytometry, activity by chromium release assay	Cortisol slope predicted survival up to 7 years later. Earlier mortality occurred among patients with relatively “flat” rhythms, indicating a lack of normal diurnal variation (Cox proportional hazards, *P* = 0.0036); associated with low counts and suppressed activity of NK cells	[4]
	43 breast cancer patients	Actigraphy, cancer-specific distress (IES, POMS), saliva samples for assessment of diurnal cortisol rhythm, cortisol awakening response (CAR), and diurnal mean. Ten potential markers of tumor progression were quantified in serum and grouped by exploratory factor analysis	Poor circadian coordination as measured by rest-activity rhythms had higher factor 1 (MMP9, TGF-beta, VEGF) scores (*R*^2^ = 0.160, *P* = 0.038). Patients with elevated CAR also had higher Factor 1 scores (*R*^2^ = 0.293, *P* = 0.020). These relationships appeared to be driven largely by VEGF concentrations	[2]
Cognitive Impairment	321 patients admitted to the Edmonton General Palliative Care Unit over a period of 26 months	Mini-Mental State Examination (MMSE) was used as screening tool to assess cognitive functioning and was performed on all patients at the time of admission and once to twice weekly thereafter	142 pts (44%) had abnormal MMSE scores (MMSE < 0.8) on admission, whereas 176 patients (55%) had abnormal MMSE scores at the time of death or discharge; 157 (68%) had abnormal MMSE scores prior to death; Of 124 patients with normal final MMSE scores, 64 (52%) were discharged versus 16 of 116 patients (14%) who had abnormal MMSE final scores (*P* < 0.0001)	[125]
	Meta-analysis of 23 studies on cognitive impairment in cancer patients	Articles published 1980-2012, comparing subjective and objective cognition in cancer patients treated with chemotherapy. Of 818 potentially relevant articles, 23 studies met the inclusion criteria for the current review and one article was sourced from reference lists of included studies	8/24 included studies found a significant relationship between objective and subjective measures of cognitive performance. These studies were more likely to involve breast cancer patients and to assess the relationship between memory and perceived cognitive impairment	[126]
	22 breast cancer survivors who reported cognitive impairment and who were at least 1 year post-chemotherapy treatment	Qualitative interviews, recorded, transcribed verbatim, and analyzed using a content analysis approach	6 major domains identified: short-term memory, long-term memory, speed of processing, attention and concentration, language and executive functioning; All survivors found these impairments frustrating, and some also reported these changes as detrimental to their self-confidence and social relationships	[127]
	85 women with early stage breast cancer scheduled for chemotherapy, 43 women scheduled for endocrine therapy and/or radiotherapy and 49 healthy control subjects	3-year prospective study; neuropsychological performance assessed at baseline (T1), post-chemotherapy (or 6 months) (T2) and at 18 months (T3)	No significant interactions or main effect of group after controlling for age and intelligence; reliable decline on multiple tasks was seen in 20% of chemotherapy patients, 26% of nonchemotherapy patients and 18% of controls at T2 (18%, 14 and 11%, respectively, at T3). Those who experienced treatment-induced menopause were more likely to show decline on multiple measures at T2 (OR = 2.6, 95%CI 0.823-8.266 *P* = 0.086)	[128]
Metabolic Abnormalities	265 patients with advanced breast cancer receiving palliative chemotherapy	Retrospective study; mortality was compared for diabetic and nondiabetic patients as well as for patients that presented hyperglycemia during treatment	Overall survival was greater in diabetic patients with proper metabolic control than diabetic patients with hyperglycemia. The risk of death was higher in patients with mean glucose levels > 130 mg/dL during treatment	[129]
	Meta-analysis of 20 studies (5 case-control and 15 cohort studies) that reported relative risk (RR) estimates (odds ratio, rate ratio/hazard ratio, or standardized incidence ratio) with 95%CI for the relation between diabetes (largely Type II diabetes) and breast cancer incidence	RRs were calculated using a random-effects model	All 20 studies showed that women with (*vs*. without) diabetes had a statistically significant 20% increased risk of breast cancer (RR, 1.20; 95%CI, 1.12-1.28). The summary estimates were similar for case-control studies (RR, 1.18; 95%CI, 1.05-1.32) and cohort studies (RR, 1.20; 95%CI, 1.11-1.30)	[130]
	Pooled individual-level data from 758,592 premenopausal women from 19 prospective cohorts	Hazard ratios (HRs) of premenopausal breast cancer in association with BMI from ages 18 through 54 years using Cox proportional hazards regression analysis. Median follow-up; 9.3 years (interquartile range, 4.9-13.5 years) per participant, with 13,082 incident cases of breast cancer	Inverse linear associations of BMI with breast cancer risk were found that were stronger for BMI at ages 18 to 24 years (HR per 5 kg/m^2^ [5.0-U] difference, 0.77; 95%CI, 0.73-0.80) than for BMI at ages 45 to 54 years (HR per 5.0-U difference, 0.88; 95%CI, 0.86-0.91). 4.2-fold risk gradient between the highest and lowest BMI categories (BMI ≥ 35.0 *vs*. 17.0) at ages 18 to 24 years (HR, 0.24; 95%CI, 0.14-0.40)	[131]
	10,786 women ages 35-69 were recruited in a prospective study in Italy; Four matched controls were chosen for each breast cancer case (*n*= 144)	Blood samples were collected after a 12-h fast between 7:30 and 9:00 a.m.	Adjusted relative risk (RR) for the highest quartile of serum glucose *vs*. the lowest was 2.8 (95%CI, 1.2-6.5), and *P* for trend was 0.02. Insulin showed a weaker association with breast cancer, the adjusted RR of the highest quartile *vs*. the lowest was 1.7 (95%CI, 0.7-4.1), and P for trend was 0.14, whereas the adjusted RR of the highest quartile of IGF-I was 3.1 (95%CI, 1.1-8.6), and P for trend was 0.01	[132]
Microbial Dysbiosis	Breast tumor tissue and paired normal adjacent tissue from the same patient	Qualitative survey of breast microbiota DNA	Bacterium *Methylobacterium radiotolerans* is relatively enriched in tumor tissue, while the bacterium *Sphingomonas yanoikuyae* is relatively enriched in paired normal tissue. The relative abundances of these two bacterial species were inversely correlated in paired normal breast tissue but not in tumor tissue, indicating that dysbiosis is associated with breast cancer	[133]
	48 postmenopausal breast cancer case patients, pretreatment, *vs*. 48 control patients	Microbiota profiles in fecal DNA were determined by Illumina sequencing and taxonomy of 16S rRNA genes. Estrogens were quantified in urine; linear and unconditional logistic regression of microbiota α-diversity (PD_whole tree) and UniFrac analysis of β-diversity	Estrogens correlated with α-diversity in control patients (Spearman Rho = 0.37, *P* = 0.009) but not case patients (Spearman Rho = 0.04, *P* = 0.77). Compared with control patients, case patients had statistically significantly altered microbiota composition (β-diversity, *P* = 0.006) and lower α-diversity (*P* = 0.004). Adjusted for estrogens and other covariates, odds ratio of cancer was 0.50 (95%CI, 0.30-0.85) per α-diversity tertile	[134]
	31 patients with early-stage breast cancer	Bacterial DNA was extracted from the feces; qPCR amplified, targeting 16S rRNA sequences specific to bacterial groups, and then analyzed in relation to clinical characteristics	Absolute numbers of total bacteria and three bacterial groups (*Firmicutes, Faecalibacterium prausnitzii*, and *Blautia*) differed significantly according to the patient's body mass index. *C. coccoides, F. prausnitzii*, and *Blautia*, differed significantly according to the clinical stages and the histoprognostic grades	[135]
	Eighteen patients with breast cancer (BC), 18 with uterine leiomyoma (UL), and 30 healthy women	Feces were collected on 1st admission and processed immediately; qualitative and quantitative analysis of fecal flora	Premenopausal BC patients showed increased Enterobacteriaceae (*E. coli*, log 9.7 ± 2.1, *P* < 0.001); aerobic streptococci (log 7.8 ± 2.0) and lactobacilli (log 8.0 ± 2.8). Anaerobic bacteria were increased (*P* < 0.001) for clostridia (log 9 ± 1.7), bacteroides (log 7.2 ± 3.1), and anaeroboic lactobacilli (9.1 ± 2.5). Similar changes in menopausal samples	[136]
Systemic Inflammation	Data from the Health, Eating, Activity, and Lifestyle (HEAL) Study (a multiethnic prospective cohort study of women diagnosed with stage 0 to IIIA breast cancer) (734 total survivors)	Concentrations of CRP and SAA were measured approximately 31 months after diagnosis and tested for associations with disease-free survival (approximately 4.1 years of follow-up) and overall survival (approximately 6.9 years of follow-up)	Elevated SAA and CRP were associated with reduced overall survival, regardless of adjustment for age, tumor stage, race, and body mass index (SAA *P* trend < 0.0001; CRP *P* trend = 0.002). The HRs for SAA and CRP tertiles suggested a threshold effect on survival, rather than a dose-response relationship (highest *vs*. lowest tertile: SAA HR = 3.15; 95%CI, 1.73-5.65; CRP HR = 2.27; 95%CI, 1.27-4.08)	[137]
	96 patients with metastatic breast cancer. During follow-up 51 patients died of their cancer	Evaluated the value of an inflammation-based score (Glasgow Prognostic Score, GPS) in patients with metastatic breast cancer (scored on 0-2 scale)	Multivariate analysis of the GPS and treatment received, only the GPS (HR 2.26, 95%CI 1.45-3.52, *P* < 0.001) remained significantly associated with cancer-specific survival	[138]
	Colorectal (*n* = 182), gastric (*n* = 87), breast (*n* = 99), or bronchogenic (*n* = 404) cancer patients, who had measurements of C-reactive protein and albumin	Median survival, univariate/multivariate analyses of correlations between inflammatory markers and survival	Association between duration of survival and both log_10_ C-reactive protein and albumin concentrations (*P* < 0.0002). log_10_ C-reactive protein was an independent predictor of survival (*P* < 0.0002). When all patients were analyzed (*n*= 772), the hazard ratio for a 10-fold increase in C-reactive protein concentration in cancer-specific survival was 2.21 (95%CI = 1.92-2.56 *P* < 0001)	[139]
	Cross-sectional and retrospective studies. CS included 100 women undergoing mastectomy for breast cancer risk reduction (*n* = 10) or treatment (*n* = 90). Retro study was 127 women who developed metastatic breast cancer	Metabolic syndrome-associated circulating factors were compared by CLS-B status. The association between CLS of the breast and the metabolic syndrome was validated; Distant recurrence-free survival (dRFS) was compared by CLS-B status	Pts with WAT inflammation had elevated insulin, glucose, leptin, triglycerides, C-reactive protein, and IL6 and lower high-density lipoprotein cholesterol and adiponectin (*P* < 0.05); Compared with patients without breast WAT inflammation, the adjusted HR for dRFS was 1.83 (95%CI, 1.07-3.13) for patients with inflammation	[140]

**Table 2.** Non-exhaustive list of primary animal model evidence for brain-tumor interactions regulating cancer incidence, disease progression, morbidity and mortality (see Figure 2 for more details)

Cancer type/model	Main focus	Primary findings	Ref.
67NR/4T1/4T07 syngeneic breast cancer cells (female BalbC mice; subQ/orthotopic)	Effects of peripheral tumors on central regulation of sleep and metabolism	Tumors alter leptin/ghrelin signaling, disrupting central hypocretin/orexin activity to influence glucose metabolism and sleep via the sympathetic nervous system	[6]
LL2 Lewis Lung carcinoma/B6 (male C57bl6j mice; subQ)	Dopaminergic regulation of tumor growth	Activation of VTA-dopamine neurons blunts tumor growth via sympathetic modulation of bone-marrow myeloid derived suppressor cells	[87]
p53^R270H©/+^WAP-Cre mutant model of Li-Fraumeni syndrome (mouse; transgenic)	Circadian disruption-induced cancer development	Chronic phase shifting accelerated spontaneous tumor growth and altered tumor phenotype	[60]
*N*-nitroso-*N*-methylurea (NMU)-induced mammary tumors (rat; chemically induced)	Effects of tumors on affective behaviors	Tumor growth is associated with central cytokine concentrations, altered glucocorticoid responses, and the development of depressive-like behavior	[141]
Colon-26 adenocarcinoma cells (mouse; SubQ)	Effects of tumors on fatigue, muscle physiology, and affective behaviors	Tumors promoted central proinflammatory cytokine production and depressive-like behavior prior to defects in muscle function, behavior rescued by SSRI	[142,143]
HeyA8, SKOV3ip1, MB-231 orthotopic human ovarian carcinoma cells (nude mice; IP)	Effects of stress on tumor development and angiogenesis	Stress-induced adrenergic signaling (cAMP->PKA) promotes tumor growth and angiogenesis	[12]
Non-metastatic methylcholanthrene-induced sarcoma (F344/NTacfBR male rats; SubQ)	Effects of inflammation on central hypocretin/orexin neurons and fatigue	Tumors reduced hypocretin/orexin transcript expression and promoted fatigue	[144]
LL2 or TC-1 lung epithelial cells (male C57Bl6 mice; subQ)	Role of sleep fragmentation (SF) on tumor growth and progression	SF accelerates tumor growth, likely through a TLR4 dependent mechanism	[13]
LL2 Lewis Lung carcinoma cells/Apc/min+ mice (male and female C57Bl6; subQ/transgenic)	Role of calcitonin-gene related peptide (CGRP) neurons in cancer-associated cachexia	Inactivation of parabrachial CGRP neurons prevents and reverses cancer-induced anorexia, fatigue, and changes in affective behavior	[109]
MADB106 breast cancer cells (outbred “hyperreactive” Wistar rats; subQ)	Role of dopaminergic system in tumor growth/metastasis	Smaller tumors, fewer metastases, and reduced angiogenesis in rats with a hyperreactive dopaminergic system	[84]
K-ras^LSL-G12D/+^;p53^flox/flox^ (KP) or K-ras^LSL-G12D/+^ (K) lung cancer model 129SvJ × C57bl6 mice (cre-dependent p53 deletion)	Effects of circadian disruption (environmental and genetic) on lung tumor growth and progression	Both genetic and physiologic circadian disruption accelerate tumor growth and promote c-myc upregulation and metabolic reprogramming	[61]
diethylnitrosamine-induced hepatocarcinogenesis (male Sprague-Dawley rats)	Sympathetic nervous system effect on hepatocarcinogenesis	High density of SNS bundles associated with poor prognosis, SNS activation of Kupffer cells drives inflammation	[145]
Hepatocarcinoma Morris 7288CTC cells (male buffalo rats) or steroid receptor (SR)-1+ or SR-1- MCF-7 human breast cancer xenografts (female nude rats)	Role of light and melatonin in cancer progression	Melatonin depleted blood accelerates tumor growth and metabolism compared to melatonin-rich blood from healthy women; light accelerates tumor growth in dose-dependent manner	[79]
B16 melanoma cells (male nude mice/C57bl6 D_2_ receptor-KO)	Role of peripheral dopaminergic signaling in tumor growth/angiogenesis/metastasis	6-OHDA ablation of dopaminergic nerves enhanced tumor angiogenesis and growth, likely through D_2_-mediated mechanism	[146]
GOS Glasgow osteosarcoma and pancreatic adenocarcinoma (P03) xenographs (male B6D2F_1_ mice; subQ into flank)	Effect of suprachiasmatic nucleus lesions on tumor growth	SCN lesions drastically increased tumor size in both cancer models examined	[147]
TC-1 mouse lung cancer cells and human lung adenocarcinoma cells (male C57bl6 mice and obstructive sleep apnea patients)	Effect of sleep fragmentation on plasma exosomes and tumor growth	Chronic sleep fragmentation alters the microRNA cargo of plasma exosomes to promote tumor cell proliferation	[148]
EG, SKOV3ip1, and 222 human ovarian cancer cells (nude male mice)	Effect of stress hormones on cancer invasiveness and growth	Adrenergic and glucocorticoid signaling promotes tumor invasiveness (in part) via upregulation of MMPs	[93]

VTA: ventral tegmental area; cAMP: cyclic adenosine monophosphate; PKA: protein kinase A; 6-OHDA: 6-hydroxydopamine; MMPs: matrix metalloproteinases
